# Vinegar Consumption Increases Insulin-Stimulated Glucose Uptake by the Forearm Muscle in Humans with Type 2 Diabetes

**DOI:** 10.1155/2015/175204

**Published:** 2015-05-06

**Authors:** Panayota Mitrou, Eleni Petsiou, Emilia Papakonstantinou, Eirini Maratou, Vaia Lambadiari, Panayiotis Dimitriadis, Filio Spanoudi, Sotirios A. Raptis, George Dimitriadis

**Affiliations:** ^1^Hellenic National Center for Research, Prevention and Treatment of Diabetes Mellitus and Its Complications (HNDC), 3 Ploutarchou Street, 10675 Athens, Greece; ^2^2nd Department of Internal Medicine and Research Institute, Athens University Medical School, Attikon University Hospital, 1 Rimini Street, 12462 Haidari, Greece; ^3^Department of Water Resources and Environmental Engineering, School of Civil Engineering, NTUA, Heroon Polytechniou 5-9, 15780 Athens, Greece

## Abstract

*Background and Aims*. Vinegar has been shown to have a glucose-lowering effect in patients with glucose abnormalities. However, the mechanisms of this effect are still obscure. The aim of this randomised, crossover study was to investigate the effect of vinegar on glucose metabolism in muscle which is the most important tissue for insulin-stimulated glucose disposal. *Materials and Methods*. Eleven subjects with DM2 consumed vinegar or placebo (at random order on two separate days, a week apart), before a mixed meal. Plasma glucose, insulin, triglycerides, nonesterified fatty acids (NEFA), and glycerol were measured preprandially and at 30–60 min for 300 min postprandially from the radial artery and from a forearm vein. Muscle blood flow was measured with strain-gauge plethysmography. Glucose uptake was calculated as the arteriovenous difference of glucose multiplied by blood flow. *Results*. Vinegar compared to placebo (1) increased forearm glucose uptake (*p* = 0.0357), (2) decreased plasma glucose (*p* = 0.0279), insulin (*p* = 0.0457), and triglycerides (*p* = 0.0439), and (3) did not change NEFA and glycerol. *Conclusions*. In DM2 vinegar reduces postprandial hyperglycaemia, hyperinsulinaemia, and hypertriglyceridaemia without affecting lipolysis. Vinegar's effect on carbohydrate metabolism may be partly accounted for by an increase in glucose uptake, demonstrating an improvement in insulin action in skeletal muscle. This trial is registered with Clinicaltrials.gov NCT02309424.

## 1. Introduction

A mixture of vinegar and olive oil is a common salad dressing used in the Mediterranean diet. The main constituent of vinegar is acetic acid, which gives vinegar its characteristic taste and smell. The consumption of vinegar with meals was used as a folk medicine for the treatment of diabetes before any pharmacologic glucose-lowering therapy [1, 2]. Recent studies indicate that vinegar improves insulin sensitivity in healthy volunteers, as well as in subjects with diabetes [3–9].

The mechanisms by which vinegar reduces glucose levels are still unclear. Acetic acid has been shown to delay gastric emptying in healthy subjects [10] and patients with type 1 diabetes [11]; alternatively, acetic acid may inhibit disaccharidase activity in the small intestine and suppress the enteral carbohydrate absorption [12]. In addition, vinegar ingestion at bedtime has been shown to decrease fasting glucose levels in humans with type 2 diabetes, suggesting an effect of acetic acid on endogenous glucose production [13]. The mechanisms of vinegar's effect on peripheral tissues have been studied in animals; these studies demonstrate that acetic acid feeding reduces glycolysis and promotes glycogen synthesis, probably by reducing xylulose 5-phosphate accumulation in the liver and phosphofructokinase-1 activity in muscle [14–16]. However, the effect of vinegar on glucose metabolism in skeletal muscle has not been studied in humans with type 2 diabetes.

In addition, previous studies indicate that acetate may also decrease circulating lipid levels [17–23] and protect from lipid accumulation in liver and skeletal muscle [24]; however, these data are derived either from animal models or from a few human studies with serious limitations.

The aim of this study was to investigate the effects of vinegar on (1) muscle glucose uptake and blood flow rates and (2) circulating plasma glucose, insulin, and lipid levels, in patients with type 2 diabetes, using the arteriovenous difference technique across the forearm muscles.

## 2. Subjects and Methods

### 2.1. Subjects

A total of eleven nonsmoking volunteers with type 2 diabetes (4 males, age 53 ± 4 years, BMI 25 ± 1, and HbA1c 6.8 ± 0.3%) participated in the study. The subjects were newly diagnosed (according to the current criteria for the diagnosis of type 2 diabetes) without drug therapy and free of diabetic complications or any other systematic disease. Their diet and body weight were stable during the last two months. All subjects were recreationally active, without any specific training programme. The subjects were instructed not to consume any acetic acid containing product for two weeks prior to the study. The study was approved by the hospital ethics committee, and subjects gave written informed consent.

### 2.2. Experimental Protocol

All subjects arrived at the hospital at 0700 h after an overnight fast and had the radial artery (A) and a contralateral antecubital vein (V) draining the forearm muscles catheterized [25, 26]. Half an hour after catheterisation, the subjects were assigned to consume vinegar (30 mL vinegar containing 6% acetic acid and 20 mL water) or placebo (50 mL water). The drinks were served at random order on two separate days which were a week apart. In each test, 5 min after the drink, the subjects consumed a meal composed of bread, cheese, turkey ham, orange juice, butter, and a cereal bar (557 kcal; 75 g carbohydrates, 26 g protein, and 17 g fat); the meal was consumed steadily within 15 min. Blood samples were withdrawn from both sides preprandially and at 15–60 min intervals for 300 min after meal for measurements of glucose (Yellow Springs Instruments, Yellow Springs, OH) and insulin (RIA; Linco Research, St. Charles, MO) and from the radial artery for measurements of triglycerides and NEFA and glycerol (Roche Diagnostics, Mannheim, Germany). A full blood count was performed preprandially.

Blood flow (BF) was measured immediately before each blood sample in the forearm with mercury strain-gauge plethysmography (Hokanson, Bellevue, WA) in the same arm as the forearm vein.

### 2.3. Calculations

Glucose plasma levels were converted to whole blood by using fractional hematocrit [27, 28]. Areas under curve were calculated by the trapezoid rule from the start of the meal to 300 min (AUC_0–300_). Glucose uptake by muscle was calculated as the arteriovenous difference of glucose multiplied by the blood flow rates [25, 26].

Results are presented as mean ± sem. Normality tests were applied to each dependent variable; all variables studied were normally distributed. Differences between the AUCs of the dependent variables were tested with paired Student's *t*-test (SPSS Inc., Chicago, IL, USA).

## 3. Results

Vinegar ingestion was well tolerated; no side-effects were reported.

### 3.1. Glucose Metabolism

#### 3.1.1. Arterial Levels of Glucose and Insulin

**Figure 1:**
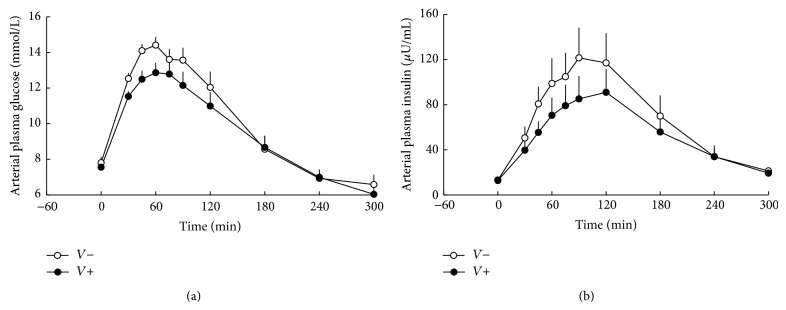
Arterial plasma glucose (*p* = 0.0279) (a) and insulin (*p* = 0.0457) (b) levels in subjects consuming vinegar (*V*+) or placebo (*V*−). At *t* = 0 min, a mixed meal was given.

Fasting blood glucose levels were similar between the two groups. In the patients who had consumed placebo, blood glucose levels raised postprandially reaching a peak after 60 min, whereas after the consumption of vinegar postprandial glucose spikes were decreased (Figure 1(a)). As a result, vinegar compared to placebo reduced total blood glucose levels (AUC_0–300 min⁡_ 2834 ± 134 versus 3005 ± 149 mM ∗ min, in vinegar and placebo group, resp., *p* = 0.0279).

Plasma insulin levels were similar between the two experiments in the fasting state. However, vinegar consumption decreased postprandial hyperinsulinaemia (AUC_0–300 min⁡_ 16136 ± 3397 versus 20473 ± 4185 mU/L ∗ min, in vinegar and placebo group, resp., *p* = 0.0457) (Figure 1(b)).

#### 3.1.2. Forearm Blood Flow

**Figure 2:**
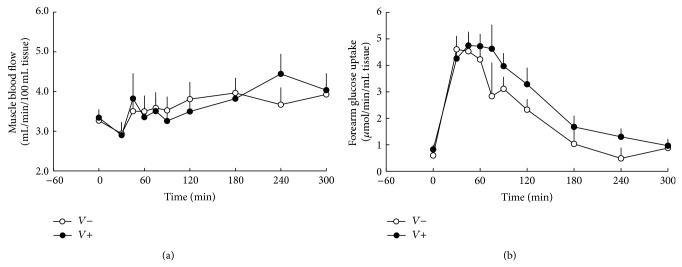
Forearm muscle glucose uptake (*p* = 0.0357) (a) and muscle blood flow (p = NS) (b) in subjects consuming vinegar (*V*+) or placebo (*V*−). At *t* = 0 min, a mixed meal was given.

Forearm blood flow rates were similar in the fasting state and remained not statistically different throughout the whole postprandial period in both groups (AUC_0–300 min⁡_ 1123 ± 73 versus 1100 ± 86 mL/min/100 mL tissue ∗ min in vinegar and placebo group, resp.) (Figure 2(a)).

### 3.2. Muscle Glucose Metabolism

In the fasting state, glucose uptake by the forearm muscles was similar in both groups. Postprandially, muscle glucose uptake was increased in the vinegar group compared to placebo (AUC_0–300 min⁡_  765 ± 87 versus 579 ± 63 *μ*mol/100 mL tissue, in vinegar and placebo group, resp., *p* = 0.0357) (Figure 2(b)).

### 3.3. Lipid Metabolism

**Figure 3:**
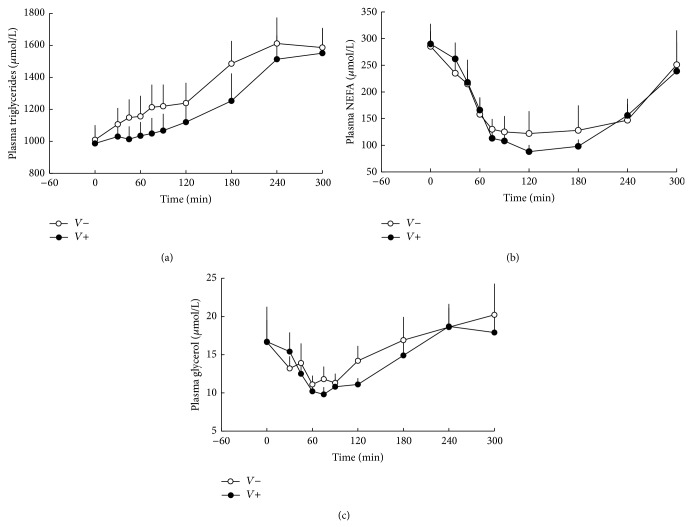
Arterial plasma triglycerides (*p* = 0.0439) (a), NEFA (p = NS) (b), and glycerol (p = NS) (c) levels in subjects consuming vinegar (*V*+) or placebo (*V*−). At *t* = 0 min, a mixed meal was given.

Fasting plasma triglyceride levels were similar between the two groups. In the vinegar group postprandial hypertriglyceridemia was less evident, resulting in decreased total plasma triglyceride levels (AUC_0–300 min⁡_  371 ± 34 versus 409 ± 38 nmol/L ∗ min, in vinegar and placebo group, resp., *p* = 0.0439) (Figure 3(a)).

Fasting plasma NEFA and glycerol levels were not different between the two groups. Postprandial plasma NEFA (AUC_0–300 min⁡_  46 ± 5 versus 49 ± 10 nmol/L ∗ min, in vinegar and placebo group, resp.) and glycerol levels (AUC_0–300 min⁡_  4 ± 0.4 versus 5 ± 0.5 nmol/L ∗ min, in vinegar and placebo group, resp.) were suppressed to the same extent between groups (Figures 3(b) and 3(c)).

## 4. Discussion

The present study investigates the effects of vinegar on circulating plasma glucose, insulin, and lipid levels, as well as blood flow rates and glucose uptake by the forearm muscles, in patients with type 2 diabetes. For this purpose we have used the arteriovenous difference technique across the forearm muscle, after the ingestion of a mixed meal, in order to create a metabolic environment which permits the interaction of insulin and substrates to be investigated under physiological conditions [25, 26, 29, 30]. To our knowledge, this is the first report examining the effect of vinegar on glucose metabolism in the skeletal muscle in humans with type 2 diabetes.

In the present study, vinegar reduced postprandial hyperglycaemia. This is supported by previous reports showing that vinegar supplementation reduces postprandial blood glucose levels in healthy subjects [2–5], as well as in subjects with insulin resistance and type 2 diabetes [7, 31]. It is also in accordance with a preliminary study reporting that regular vinegar ingestion reduces haemoglobin A1c values in patients with type 2 diabetes [32]. However, our results are not in agreement with a previous report showing that vinegar ingestion before an oral glucose load did not improve oral glucose tolerance in patients with type 2 diabetes [33]. These discrepancies could be explained, at least in part, by differences in the form of acetic acid, as well as the kind of the test meal following acetic acid ingestion. As shown previously, acetic acid reduced postprandial glucose values when it was administered in the form of vinegar, but not in the form of sodium acetate [34]. In addition, the glucose-lowering effect of vinegar was evident when vinegar was ingested with complex carbohydrates, but not with monosaccharides [3, 33]. Moreover, a previous study showed that vinegar reduced postprandial glycaemia in patients with type 2 diabetes when added to a high, but not to a low, glycaemic index meal [31].

Glucose regulation depends mainly on insulin secretion by the pancreatic beta-cells and insulin action on peripheral tissues. In our study, insulin levels were decreased after the consumption of vinegar, confirming previous reports [5, 7], suggesting that the hypoglycemic effect of vinegar may be mediated through an effect on insulin action in the peripheral tissues. Skeletal muscle is considered as the most important tissue for insulin-stimulated glucose uptake [35]. In our study vinegar ingestion enhanced glucose disposal, suggesting an improvement in insulin action in skeletal muscle. It is well known that insulin affects vascular endothelium and increases muscle and adipose tissue blood flow by increasing vasodilation and capillary recruitment [35–37]. This effect is considered as an important component of insulin's stimulation of glucose uptake; impairment of this mechanism in insulin-sensitive tissues may partly account for insulin resistance in obesity and type 2 diabetes [29, 30]. Previous studies in nondiabetic humans suggest that vinegar ingestion may enhance flow-mediated vasodilation through endothelial nitric oxide synthase phosphorylation [38]. In addition, our previously published data on the effects of vinegar on muscle blood flow in subjects with impaired glucose tolerance have shown that vinegar ingestion before a mixed meal results in an enhancement of muscle blood flow rates after the meal, although postprandial insulin levels were decreased compared to their respective values in the group consuming placebo [39]. However, in our study vinegar ingestion did not alter muscle blood flow rates, suggesting that the increase in glucose disposal after meal ingestion may not be attributed to a direct effect of vinegar on blood flow in subjects with type 2 diabetes. A possible explanation for this discrepancy between individuals with impaired glucose tolerance and patients with type 2 diabetes could be that in the early stages of glucose intolerance the defect of blood flow may be reversible; however in overt type 2 diabetes the defect in flow-mediated vasodilatation may be already established and cannot be reversed by vinegar ingestion.

The effect of vinegar in the intracellular pathways of glucose metabolism in skeletal muscle has been previously examined in animal studies. In rats, acetic acid has been shown to enhance glycogen repletion, attributed to accumulation of glucose 6-phosphate due to suppression of glycolysis [14–16]. The same effect has been reported in horses after exercise. In these studies acetate supplementation enhanced the rate of muscle glycogen resynthesis during the first hours following the exercise period compared with the control treatment [40]. Although the intracellular pathways of glucose metabolism were not investigated in our study, these in vitro studies suggest that the increase in glucose uptake by the skeletal muscle following vinegar ingestion could be explained by increased rates of glycogen synthesis.

In our study, vinegar ingestion decreased postprandial hypertriglyceridaemia, without affecting NEFA and glycerol; to our knowledge, this is the first study investigating the acute effects of vinegar on lipid metabolism in subjects with type 2 diabetes. The effect of vinegar on lipid metabolism has been investigated in several studies showing that chronic administration of acetic acid reduces serum and hepatic triglyceride levels [17, 19, 41] in metabolically healthy animals. In addition, chronically administered acetate treatment in obese [18] and/or type 2 diabetic [21] rats has been shown to result in a reduction of plasma triglyceride levels. In contrast, triglyceride levels were not affected by acute administration of 10 mL vinegar added to a hypercholesterolaemic diet in rabbits [20]. On the other hand, information on humans is limited to a few studies examining the chronic effect of vinegar with conflicting results. In these studies 15–30 mL vinegar intake for 8–12 weeks resulted in a decrease of serum triglyceride levels in subjects with obesity [23] or hyperlipidaemia [22]. However, there was no effect of vinegar intake in a prospective randomized, double blind, placebo-controlled clinical study conducted in 114 nondiabetic subjects consuming 30 mL apple vinegar for 8 weeks [42]. The results of this study should however be considered with caution since this study had several limitations; the most important is the mixed group of subjects (one-third of the participants were on statin and/or fish oil treatment).

Previous animal studies suggest that the effect of vinegar on triglyceride levels could be attributed to the inhibition of hepatic lipogenesis and increase of fatty acid oxidation [17, 21]. However, this mechanism could not explain the results of the present study, since vinegar ingestion had no acute effect on plasma levels of NEFA and glycerol. As a result, although chronic administration of vinegar could have an impact on fatty acid metabolism [21, 23] our study showed that the acute administration of vinegar in subjects with type 2 diabetes does not affect lipolysis. A possible explanation of these findings could be that the acute intake of vinegar increases insulin sensitivity of the adipose tissue, increasing the lipoprotein lipase activity and the postprandial clearance of triglycerides [28, 30], with no effect on hormone-sensitive lipase, which regulates lipolysis.

Although the arteriovenous difference technique has allowed insights into the glucose fluxes across the forearm muscles, some limitations should be considered when interpreting the results. First, the number of participants was relatively small. This was mainly due to the invasive nature of the technique and the need for repeating the experiment after one week. However, due to the crossover design of the study, our data were sufficient for reaching statistical significance. Moreover, another limitation of our study was that the patients had mild diabetes (HbA1c: 6.8 ± 0.2%). This was due to the fact that we aimed to see the effect of vinegar in patients with newly diagnosed diabetes, without diabetic complications and without taking any medication therapy that could affect glucose or lipid metabolism. Further studies are needed to investigate the effect of vinegar on glucose metabolism in patients with more severe type 2 diabetes, as well as in those taking various treatments.

In summary, our study showed that, in type 2 diabetes, vinegar reduces postprandial hyperglycaemia, hyperinsulinaemia, and hypertriglyceridaemia without affecting lipolysis. As a result, vinegar's effect on carbohydrate metabolism may be accounted for, at least in part, by an increase in insulin-stimulated glucose uptake, demonstrating an improvement in insulin action in the skeletal muscles. However, further studies are required to examine the long-term effects of vinegar in type 2 diabetes.
